# A multicenter study of endocrine abnormalities in septo-optic dysplasia (SOD) in Asean countries

**DOI:** 10.1186/1687-9856-2015-S1-P85

**Published:** 2015-04-28

**Authors:** Suttipong Wacharasindhu, Pattareeya Yottasan, Somchit Jaruratanasirikul, Ouyporn Panamonta, Kevalee Unachak, Aman Pulungan, Muhammad Faizi, Nur Rochmah, Susana Campos, SiokSoan Chan Cua, Fatimah Binti Harun, Muhammad Yazid Jalaludin, Vu Chi Dung Noi tiet

**Affiliations:** 1 A ASEAN Study group, Thailand; 2 A ASEAN Study group, Indonesia; 3 A ASEAN Study group, Philippines; 4 A ASEAN Study group, Malaysia; 5 A ASEAN Study group, Vietnam

## Background

Septo-optic dysplasia (SOD) is a heterogeneous malformation condition consisting of optic nerve hypoplasia, various types of forebrain defects and hormonal deficiencies. This study aims to expand knowledge about endocrine abnormalities in patients with SOD in ASEAN countries.

## Material and method

Forty-eight patients (27 male, 21 female) who has been diagnosed as having SOD in ASEAN countries were clinically reviewed from medical records.

## Results

Clinical manifestations and endocrine abnormalities of the patients are shown in Table 1

**Table 1:** Clinical manifestation of SOD patients

Clinical manifestation			Clinical manifestation		
Age at presentation (months) mean ± SD		33 ± 39 months (range 20 - 178)	MRI findings	Absence of septum pellucidum(%)	62
		
Ht SDS, mean ± SD		-0.97 ± 1.97		Pituitary hypoplasia (%)	33
		
Wt SDS, mean ± SD		0.02 ± 2.35		Abnormal of corpus callosum (%)	16
		
Eye presentation	Nystagmus (%)	54		Cortical dysplasia (%)	8
	
	Squint (%)	3	Endocrine abnormalities	Hypothyroid (%)	35
			
	Poor vision (%)	35		GH insufficiency (%)	29
		
Delayed development (%)		16.2		Delayed/arrested puberty/suspected HG (%)	23
		
Seizure (%)		7.9		Cortical insufficiency (%)	17
		
Hypoglycemia (%)		8.1		Diabetes insipidus (%)	19

Neonatal jaundice (%)		2.7	Eye examination	Unilat ON hypoplasia	13
		
Undescended testes (%) (boys)		5.4		Bilat ON hypoplasia	84
		
Micropenis (%) (boys)		21		Microcornea	3

HG = Hypogonadism, ON = Optic nerve

## Conclusion

This multicenter and multinational study shows that about 20-35 % of SOD patients have endocrine abnormalities. Hypothyroidism and GH insufficiency are the most common endocrine problems associated with this condition.

